# Influence of Drilling Strategy and Cutting Parameters on Selected Aspects of the Single-Shot Drilling of CFRP/AISI 316L Steel Stacks

**DOI:** 10.3390/ma19122546

**Published:** 2026-06-12

**Authors:** Krzysztof Szwajka, Joanna Zielińska-Szwajka, Tomasz Trzepieciński, Marek Szewczyk

**Affiliations:** 1 Department of Integrated Design and Tribology Systems, Faculty of Mechanics and Technology, Rzeszów University of Technology, ul. Kwiatkowskiego 4, 37-450 Stalowa Wola, Poland; m.szewczyk@prz.edu.pl; 2 Department of Component Manufacturing and Production Organization, Faculty of Mechanics and Technology, Rzeszów University of Technology, ul. Kwiatkowskiego 4, 37-450 Stalowa Wola, Poland; j.zielinska@prz.edu.pl; 3 Department of Manufacturing Processes and Production Engineering, Rzeszów University of Technology, al. Powstańców Warszawy 8, 35-029 Rzeszów, Poland; tomtrz@prz.edu.pl

**Keywords:** composite/metal stack materials, drilling, drilling strategy, single-shot drilling, machining parameters, delamination, temperature, surface quality

## Abstract

Drilling is often used to create holes in CFRP/AISI 316L hybrid stacks to facilitate the assembly process. Due to the non-uniform properties and difficult machinability of Cr-Ni-Mo AISI 316L steel, drilling CFRP/AISI 316L stacks poses significant challenges in manufacturing processes. This paper aims to evaluate the tool–workpiece interaction and the effect of the drilling strategy on the technological aspects of drilling CFRP/AISI 316L stacks. The experimental results show that cutting parameters have a significant impact on the drilling performance of CFRP/AISI 316L stacks. The AISI 316L → CFRP drilling strategy provides lower hole surface roughness with less burr formation in the AISI 316L layer, while the CFRP → AISI 316L drilling strategy is preferred in terms of minimizing delamination damage. The high temperature generated during drilling of the AISI 316L layer directly affects the hole surface quality in the CFRP layer and the phenomena occurring in the interlayer of the stack materials. The experimental results presented in this work allowed us to formulate several recommendations regarding the selection of cutting strategy and cutting parameters when drilling CFRP/AISI 316L hybrid stacks.

## 1. Introduction

Among the laminated materials widely used in the modern marine industry, CFRP/stainless steel laminates are the most popular. Key applications for stainless steel-based fiber metal laminates (FMLs) include deck structures, hulls, and masts. The greatest advantage of combining carbon fiber-reinforced polymers (CFRPs) and stainless steel in the lamination process is improved overall strength while providing high resistance to seawater [1]. The 316L stainless steel sheets are one of the most popular choices in the marine industry due to their exceptional durability, corrosion resistance, and high strength [2,3]. Stainless steel-based composite materials are mainly used for marine applications due to their high strength and anti-corrosion properties [4]. CFRP/316L hybrid materials make components significantly lighter, more durable, and resistant to extreme marine conditions [5]. This allows ships that use wood-based [6,7] and metal-based [8] structures with carbon fiber reinforcement to sail faster while using less fuel.

Temporary and permanent fastenings are essential for connecting vessel structures [1,9]. Bolted or riveted connections offer advantages such as high reliability, ease of assembly, and ease of inspection. Drilling a large number of through holes is necessary to create connections. To improve component integrity, reliability, and secure assembly with other components, hole drilling is a traditional machining method in the manufacturing sector [10,11,12]. To reduce hole positioning errors and dimensional tolerances, CFRP/stainless steel sheets are single-shot drilled in industrial practice. Single-shot drilling of CFRP/metal allows for minimizing positioning errors [13]. CFRP is an anisotropic material, and its polymer matrix and carbon fiber have very different physical and mechanical properties. Due to the significant differences in the mechanical and thermophysical properties of the layers, FMLs pose challenges in machining. The discontinuity of CFRP/stainless steel stacks and their properties increases the difficulty of selecting the cutting tool profile and cutting process parameters.

The poor thermal conductivity and low interlayer strength of CFRP together lead to a complex cutting mechanism and susceptibility to defect formation [14]. The main defects occurring during drilling FMLs include fiber breakage [15,16], fiber delamination [17,18], matrix aging [19], and exit burr formation [20,21]. Push-out delamination is caused by thrust force inserted by the drill point [22], whereas peel-up delamination is caused by tool geometry [23]. Delamination significantly reduces fatigue life [24] and reduces the load-bearing capacity and tensile strength [25] of components. To reduce fiber pull-out, tearing, and delamination, numerous experimental and numerical studies have been conducted, yielding favorable results [26]. Ashrafi et al. [27] found that increasing the feed rate had a positive effect on entrance delamination in drilling CFRP-Al stacks. Wang et al. [28] reported that increasing the axial feed rate in drilling CFRP/7075 aluminum alloy stacks reduces contact time and friction and promotes faster chip evacuation. Multi-material stack (MMS) drilling poses a challenge due to burr formation, which causes stress concentration [29]. MMS drilling is also problematic due to short tool life, as multi-layer stack materials exhibit completely different machining characteristics than solid single-layer materials [30]. The exit burr is formed by plastic deformation of the workpiece material in front of the chisel edge. The burr formed at the entrance is caused by tearing and is smaller than the exit burr height [31]. Mathawan et al. [11] emphasized the influence of drill geometric parameters on hole quality when drilling CFRP/7075-T6 aluminum alloy stacks. In addition to conventional machining methods, alternative non-traditional technologies such as laser-assisted drilling [32], abrasive waterjet drilling [33], orbital drilling [34], and tilting helical milling [35] are being developed to offer low-damage solutions. Ultrasonic vibration-assisted machining, which promotes tool–chip separation [36], has been proposed to improve hole quality while reducing heat. Xu et al. [37] analyzed hole inlet and outlet surface morphology, tool wear, and cutting force in ultrasonic-assisted drilling (UAD) of CFRP/Ti stacks. It was reported that UAD improved hole surface morphology and reduced the damage coefficient by 15.7% compared to conventional drilling. According to Wang et al. [14], high-performance coatings and variable feed strategies prevent burrs and delamination. A systematic explanation of the damage mechanism has not yet been developed [14]. Non-conventional drilling methods offer micro-hole machining, zero tool wear, micro-hole machining, deburring, and low-damage hole-making in CFRP. Current research efforts are focused on developing real-time, in situ intelligent monitoring systems to quantify defect formation and reveal underlying material removal mechanisms.

The quality of composite components is related not only to mechanical damage to the hole surface but also to thermal damage associated with the heat generated during drilling [38]. Degradation of the resin matrix in CFRP occurs after exceeding the glass transition temperature (approximately 180 °C) [39,40]. The significance of heat-induced effects on hole quality increases with increasing metal layer thickness in FMLs. During metal drilling, a large amount of heat is generated and accumulates in the stack contact area. Reduced heat dissipation intensifies heat-induced phenomena that reduce material strength. Therefore, processing temperature testing, in addition to assessing the hole surface quality, is crucial to ensuring adequate cutting quality. Studies by Kim et al. [41] and Ramulu et al. [42] confirmed that thermal damage to the composite is more severe during stack drilling; therefore, many researchers first focus on the drilling temperature when drilling stacks [28].

Current research on the cutting mechanism of laminated structures is based primarily on the assessment of microscopic accuracy and surface integrity after machining. The vast majority of studies concern the machining of CFRP-based FMLs used in the aerospace industry, including laminates combining CFRP and light metal alloys, such as aluminum, titanium, and their alloys. Isbilir and Ghassemieh [43] proposed a model of interlaminar delamination that uses a coherent contact area to describe the occurrence and propagation of delamination. Li et al. [44] analyzed the strain evolution during drilling of CFRP/Ti stacks. They found that under conditions of low axial feed rate and low spindle speed, the hole quality in the stack is higher. To improve the surface quality of polymer/metal stacks, a better understanding of drilling mechanisms plays a key role in optimizing tool geometry and cutting parameters. Layer delamination during drilling CFRP/Ti6Al4V stacks decreases with cutting speed but increases with axial feed rate [45]. Performance studies of various modified twist drills showed that both point angles and helix angles were key factors influencing the drilling performance of CFRP/Ti6Al4V stacks [46]. Zhang et al. [47] proposed a variable-parameter drilling method for Ti-CFRP-Ti stacks. It was demonstrated that the optimized drilling parameters can improve the drilling efficiency and effectively reduce the hole wall surface roughness. In summary, to ensure the integrity of drilled hole surfaces, optimal cutting parameters must be selected to minimize machining errors and the effect of heat on the properties of the stack materials, particularly CFRP. Although most existing studies focus on optimizing parameters in conventional drilling, Yang et al. [48] investigated the effect of drill bit design (dagger drill, drill bit, candlestick drill, and twist drill) on the interlaminar drilling performance of CFRPs. Studies on the morphological characteristics of material damage revealed that twist drills are more suitable for interlaminar drilling in CFRPs.

Currently, articles discussing drilling techniques in CFRP-based FMLs mainly focus on drilling the following stacks: CFRP/titanium, CFRP/titanium alloy, CFRP/aluminum, and CFRP/aluminum alloy. Research on the effect of machining parameters on hole surface integrity in CFRP/stainless steel stacks is an unexplored area. Few studies address drilling CFRP/carbon steel stacks. Kolesnyj et al. [49] investigated the effect of cutting temperature on the tool life of carbide drills in drilling CFRP/S235J0 stacks. These authors reported that the evolution of cutting temperature in drilling CFRP/metal stacks has not yet been fully understood. Research on drilling temperature in stacks, especially in the contact area, is still insufficient because heat transfer is complex due to discontinuous heat conduction [38]. In the context of improving drilled hole quality, Bleicher et al. [50] proposed vibration-assisted machining at low frequencies when drilling CFRP/C45E stacks. Buescart et al. [51] analyzed the hole quality of robotically drilled glass fiber-reinforced polymer/AISI 304 stacks. Due to the unexplored area of drilling stainless steel-based FMLs, this article presents the results of single-shot drilling of CFRP/AISI 316L stacks. The aim of the study was to determine the tool–workpiece interaction and the impact of cutting sequence strategies (AISI 316L → CFRP and CFRP → AISI 316L) on technological aspects of stack cutting, including cutting resistance, destruction mechanisms, hole surface quality, chip morphology, and chip temperature. Based on an extensive research campaign, recommendations were formulated regarding the selection of cutting sequence strategy and cutting parameters when drilling CFRP/AISI 316L hybrid stacks.

## 2. Materials and Methods

### 2.1. Workpiece Material

**Figure 1:**
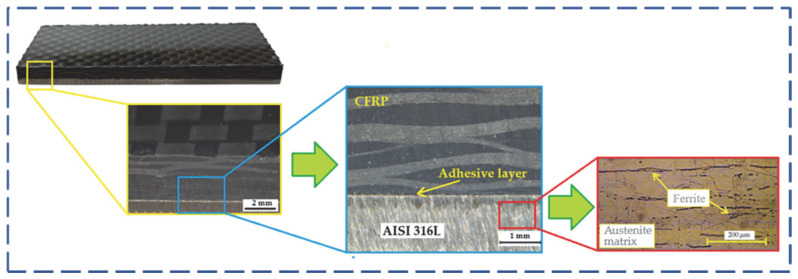
The structure of the materials used to produce stacks.

The hybrid stack used for the drilling experiments consisted of a CFRP laminate (DEXCRAFT, Warsaw, Poland) and a Cr-Ni-Mo AISI 316L steel plate. AISI 316L steel is designated X2CrNiMo17-12-2 (1.4404) according to European standards and belongs to the group of austenitic steels. The thicknesses of the CFRP laminate and AISI 316L plate were 3 mm and 1.5 mm, respectively. The CFRP laminate was bonded on one side to the surface of the AISI 316L plate (Figure 1). In the CFRP laminate, the fibers in a carbon fabric with a plain weave run at an angle of 0°/90° to each other. As shown in Figure 1, the plain weave is a pattern where interwoven longitudinal fibers (warp) cross over transverse fibers (filling) in an alternating fashion, resulting in a consistent and symmetrical cross pattern. A single carbon fiber has a diameter of approximately 7 μm. The various physical and mechanical properties of the materials used in the hybrid stack directly impact the drilling process. Selected physical and mechanical properties of the stack materials are listed in Table 1.

Hybrid stacks were prepared for testing in a form of strips 30 mm wide and 500 mm long. A strip of AISI 316L sheet was bonded to a CFRP laminate using a ACRA LOCK SA 10–15 BLK methacrylate adhesive (Engineered Bonding Solutions, Titusville, FL, USA). Before applying the adhesive, the surfaces of the steel plate and CFRP laminate were ground. The bonded surfaces were then cleaned with isopropyl alcohol. In the bonding process, the adhesive was first applied evenly to the AISI 316L plate surface using an applicator and then to the surface of the CFRP laminate. After applying the adhesive, the bonded materials were pressed together to ensure the required conditions for a sound joint. The bonding parameters were as follows: temperature 22 ± 1 °C, pressure 50 N/mm^2^, and handling time 6 h.

### 2.2. Cutting Tool

**Figure 2:**
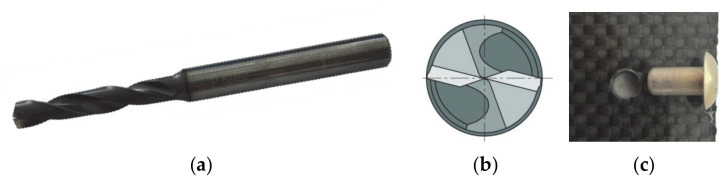
View of (**a**) the drill and (**b**) its cutting edges; (**c**) a typical rivet.

The cutting tool used in the drilling tests was a TiAlN-coated cemented carbide drill (Figure 2a,b) from Iscar Poland Sp. z o.o. (Katowice, Poland). The designation of the drill is SCD 048-020-060 AP3N IC908. The IC908 is a submicron PVD-coated grade recommended for general use in a variety of operations with materials such as alloy steels, austenitic stainless steel, and heat-resistant alloys at low-to-medium cutting speeds. It is characterized by high wear resistance. The drill’s geometric parameters are as follows: the drill bit size is 4.8 m, the helix angle is 30°, the point angle is 140°, the chisel edge length is 0.14 mm, and the point length is 0.8 mm. The choice of the drill diameter used was dictated by the diameter of a typical rivet with a diameter of 4.76 mm (3/16″) (Figure 2c).

### 2.3. Equipment and Machining Conditions

**Figure 3:**
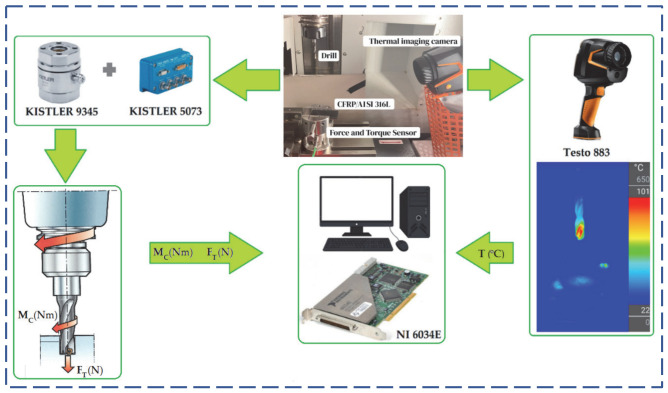
Diagram of experimental setup and schematic of the DAS.

A schematic diagram of the experimental setup and a schematic of the data acquisition system (DAQ) are shown in Figure 3. The drilling experiments were performed on a Concept Mill CNC vertical milling machine (EMCO GmbH, Hallein, Austria). Particular attention was paid to the influence of the drilling sequence (CFRP → AISI 316L and AISI 316L → CFRP) on the machining characteristics and damage mechanisms of the CFRP/AISI 316L stacks at different cutting parameters (Table 2). All drilling experiments were conducted under dry cutting conditions.

While an Iscar submicron cemented carbide drill with a TiAlN PVD coating is excellent for austenitic stainless steel, TiAlN coatings are known to suffer from rapid abrasive wear when encountering carbon fibers, which can lead to micro-chipping of the cutting edge. In the drilling experiments, each drill was used to drill only one hole to exclude the influence of tool wear on the hole damage in drilling. The aim of this strategy was to minimize the impact of the wear-out TiAlN coating on the drilling results of CFRP/AISI 316L stacks. Furthermore, the experiments for each set of cutting parameters were repeated three times to obtain the arithmetic mean value of the quantitative results. Table 2 presents the machining parameters adopted in the drilling tests. It should be noted that the drilling parameters were selected based on the data recommended by the cutting tool manufacturer for drilling CFRP and AISI 316L steel. Drill bit and chip temperatures were measured using a Testo 883 (Testo SE & Co. KGaA, Titisee-Neustadt, Germany) thermal imaging camera, as shown in Figure 3.

Thrust force (F_T_) and cutting torque (M_C_) signals were measured using a KISTLER^®^ 9345B piezoelectric sensor (KISTLER, Winterthur, Switzerland). The signals from the sensors were recorded on a personal computer’s hard drive via a National Instruments^®^ PCI-6034E multifunction data acquisition card (Austin, TX, USA). The sampling rate of the signals during the experiments is 50 kHz, and the card’s sampling accuracy is 16 bits.

## 3. Results and Discussion

### 3.1. Cutting Resistance

**Figure 4:**
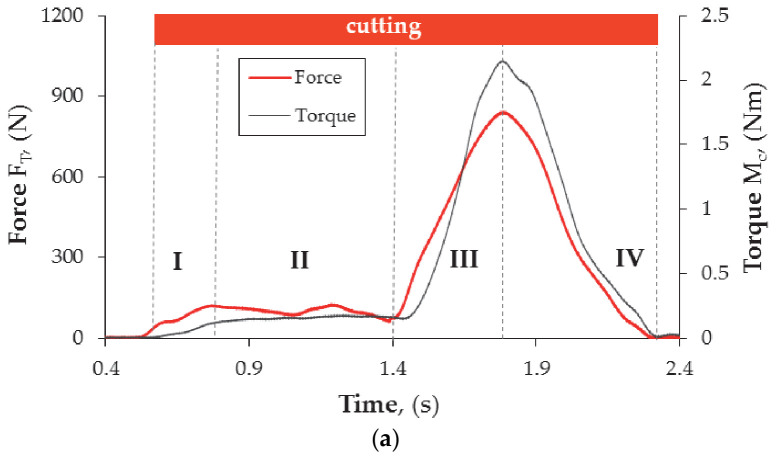

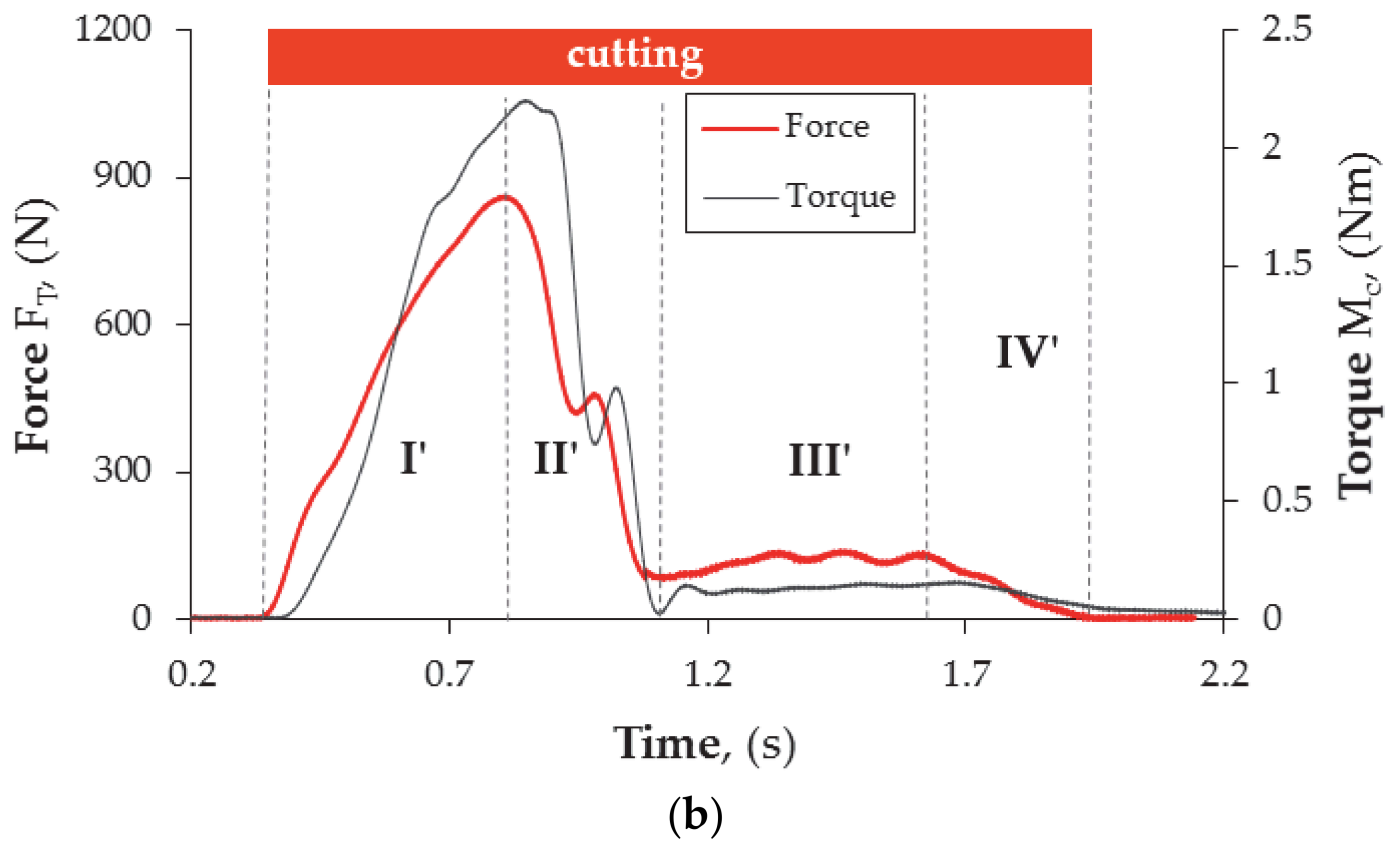
Variation of thrust force and cutting torque signals when drilling CFRP/AISI 316L stacks for cutting speed v_c_ = 15 m/min, axial feed rate f = 0.15 mm/rev, and different drilling strategies: (**a**) CFRP → AISI 316L and (**b**) AISI 316L → CFRP.

A repeatable pattern was observed in the thrust force (F_T_) and cutting torque (M_C_) signals as a function of cutting time. The only significant difference in these signals is related to the drilling strategy (CFRP → AISI 316L, AISI 316L → CFRP). Figure 4 shows the variation of the thrust force and cutting torque signals during drilling of stacks with a cutting speed of v_c_ = 15 m/min and an axial feed rate of f = 0.15 mm/rev. For the CFRP → AISI 316L drilling strategy, four characteristic stages were observed during the machining of a single hole, concerning the variation of both the axial force and cutting torque signals. In the initial phase (stage I) of drilling for the CFRP → AISI 316L strategy, the chisel edge of the drill enters the CFRP layer. At this stage, the values of both the axial force and the cutting torque increase.

The cutting force and torque increase until both cutting edges of the drill have penetrated the workpiece. In stage II, the cutting edges of the drill have completely penetrated the CFRP material, and the thrust force has stabilized, reaching a maximum of 120 N. At the beginning of stage III, the drill begins cutting through the AISI 316L layer. As the chisel edge of a drill begins to penetrate the AISI 316L plate (stage III), the thrust force increases rapidly until the cutting edges of the drill have completely penetrated the AISI 316L material, reaching a maximum value of approximately 850 N at the end of stage III. In stage IV, the thrust force rapidly decreases. No stable area was observed in this stage where the thrust force stabilized for a longer period. This is due to the fact that the thickness of the AISI 316L sheet was half that of the CFRP layer. Taking into account the drill point length, the drill’s cutting edges cut the full cross-section of the AISI 316L layer in half the time required for CFRP. The thrust force value when drilling AISI 316L increased sevenfold compared to drilling through the CFRP layer. The end of stage IV corresponds to the moment the drill exits the processed stack. It is difficult to determine from the thrust force signal which stage is clearly related to the processing of the adhesive layer connecting the CFRP and AISI 316L layers. This means that no gap occurred at the interface between the stack materials. In general, it is important to avoid situations where chips generated during the drilling process of the AISI 316L plate could be pressed into the interlayer gap between the CFRP and AISI 316L layers.

For the AISI 316L → CFRP drilling strategy, it was also observed that during the entire drilling process, four characteristic stages occurred, concerning the variation of both the thrust force signal and the cutting torque (Figure 4b). In the initial stage of the drilling process (stage I′), the chisel edge of the drill penetrates the AISI 316L layer. The thrust force value increases rapidly in this stage. However, there is no phase in which the thrust force value stabilizes. This is due to the above-described difference in the thicknesses of the CFRP and AISI 316L layers. The thrust force reached a maximum value of approximately 820 N in this stage. At the beginning of stage III′, the drill begins to penetrate the CFRP layer. During stage III′, the cutting edges of the drill completely penetrated the CFRP layer, and the thrust force value stabilized, reaching a maximum value of 140 N during this stage. During stage IV′, a rapid decrease in the thrust force was observed. The thrust force increased more than fivefold compared to drilling in a CFRP laminate. The end of stage IV′ corresponds to the moment the drill exits the workpiece.

The cutting torque signal (M_c_) exhibited a similar variability to the axial force (Figure 4b). As the chisel edge of a drill penetrates the workpiece layers, the cutting edge of the drill is closer to the drill axis, resulting in a lower cutting torque. Therefore, the rate of increase in the cutting torque was slower than that observed for the thrust force.

**Figure 5:**
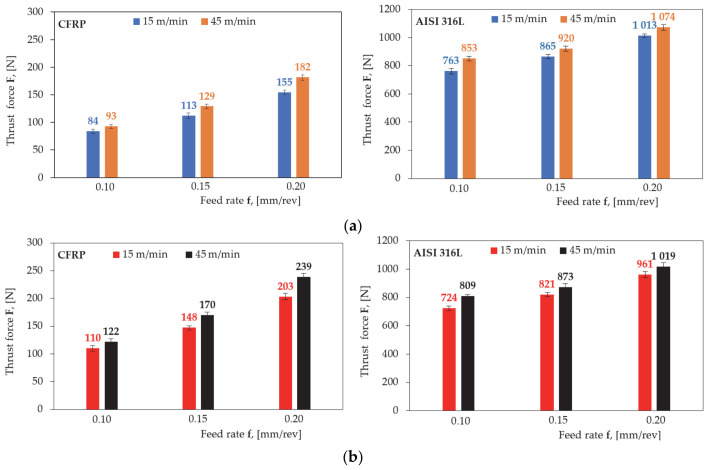
Influence of axial feed rate and cutting speed on the mean thrust force F_Tmean_ when drilling CFRP/AISI 316L stacks with different drilling strategies: (**a**) CFRP → AISI 316L and (**b**) ANSI 316L → CFRP.

Figure 5 shows the evolution of the mean thrust force F_Tmean_ for different drilling strategies and changes in axial feed rate and cutting speed. The mean thrust force and cutting torque were determined based on the signal values for the corresponding drilling steps explained in Figure 4. In all cases, a similar trend was observed regarding the changes in both thrust force and cutting torque depending on the drilling strategy adopted. When drilling the AISI 316L plate using the CFRP → AISI 316L drilling strategy, an increase of approximately 4% in the mean thrust force was observed compared to the AISI 316L → CFRP drilling strategy. A similar situation was observed when drilling the CFRP layer with the AISI 316L → CFRP drilling strategy. A 17% increase in the mean thrust force was observed compared to the mean thrust force obtained for the CFRP → AISI 316L drilling strategy.

**Figure 6:**
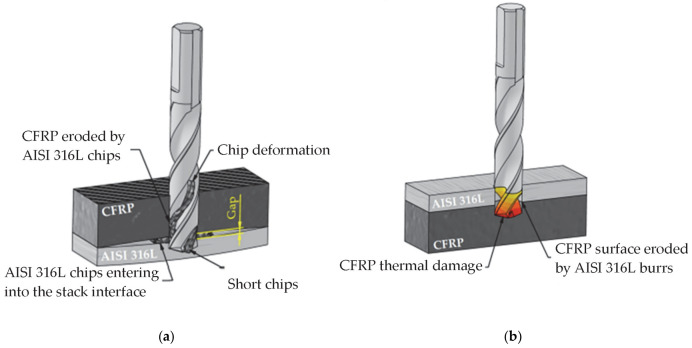
Main mechanisms of hole surface deterioration with different drilling strategies: (**a**) CFRP → AISI 316L, (**b**) AISI 316L → CFRP.

As previously stated, the thrust force when drilling AISI 316L material for the CFRP → AISI 316L drilling strategy reaches a higher value compared to drilling with the AISI 316L → CFRP strategy. The probable reason is that the evacuation process of AISI 316L chips is hindered (Figure 6a), which consequently leads to an increase in the thrust force value. AISI 316L chips rub against the drilled hole surface in the CFRP layer, which is why they are subjected to compression, thus increasing the thrust force value. When drilling the AISI 316L plate with the AISI 316L → CFRP strategy, the chips are quickly evacuated outward through the chip flutes of the drill. Moreover, in the CFRP → AISI 316L drilling sequence, interlayer delamination between both materials may occur (Figure 6a), caused by a slight elastic deflection of the AISI 316L material, which consequently leads to an increase in the thrust force. Moreover, increasing the interlayer gap width eases the ingress of steel chips into the stack interface [52].

Furthermore, cutting temperature also has a significant impact on changes in the thrust force value occurring during drilling, especially in the context of machining difficult-to-cut materials. Temperature changes can affect the properties of the workpiece material as well as the properties of the tool itself, which consequently translates into changes in the thrust force value [53]. The effect of temperature on thrust force in the drilling process of AISI 316L steel is a complex phenomenon. In the CFRP → AISI 316L drilling strategy, as the drill passed through the CFRP layer, its temperature increased, resulting in a higher initial drill temperature at the beginning of drilling in the AISI 316L layer. For the CFRP → AISI 316L drilling strategy, the thrust force value for drilling in the CFRP layer is lower than during drilling using the AISI 316L → CFRP strategy. This may result in the burning of the epoxy resin layer of the CFRP on the hole surface (Figure 6b) due to the higher drill temperature (above 190 °C). This high temperature causes resin oxidation, which is indirectly related to increased cutting resistance. Furthermore, an increase in the thrust force was observed with increasing cutting speed. AISI 316L steel is a difficult-to-cut material due to its increased nickel content and the addition of molybdenum. These elements increase corrosion resistance, particularly to chlorides (i.e., salt water), but reduce machinability. One of the main factors affecting the machinability of AISI 316L steel is its strong tendency to work hardening [54].

**Figure 7:**
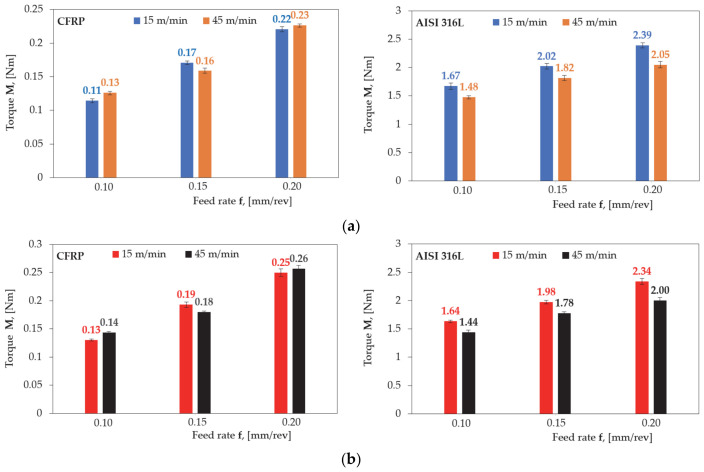
The effect of axial feed rate and cutting speed on cutting torque when drilling a CFRP/ANSI 316L stack with different drilling strategies: (**a**) CFRP → ANSI 316L and (**b**) ANSI 316L → CFRP.

In the case of mean cutting torque M_cmean_ (Figure 7), similarly to the case of thrust force (Figure 5), trends were observed regarding the influence of drilling strategy and cutting parameters on the change in cutting torque. In the case of drilling in the AISI 316L layer in the CFRP → AISI 316L strategy, for both analyzed cutting speeds, an increase in cutting torque value by approximately 4% was observed compared to the AISI 316L → CFRP drilling strategy. However, the opposite relation was observed in the case of drilling in the CFRP layer. In the AISI 316L → CFRP drilling strategy, an approximately 12% increase in cutting torque value was noted compared to the CFRP → AISI 316L strategy.

As previously stated, the cutting torque value when drilling the AISI 316L layer with the AISI 316L → CFRP strategy is slightly lower than when drilling with the CFRP → AISI 316L strategy. The difficult chip removal process of the AISI 316L plate led to a certain increase in drilling temperature and cutting torque, similar to the thrust force. As AISI 316L chips are evacuated from the hole, they can interact with the CFRP layer in the CFRP → AISI 316L drilling strategy. When drilling the CFRP layer with the AISI 316L → CFRP strategy, the fine powder chips generated during cutting the CFRP layer do not affect the machined surface of the hole in the AISI 316L layer.

### 3.2. Chip Morphology

The shape of a chip results from phenomena occurring in the cutting zone and, therefore, has a significant impact on the cutting process. A chip can be difficult to remove from the machining zone, entangling the tool or workpiece and affecting the hole surface. Furthermore, it is crucial that the chip quickly exits the cutting zone. Three basic chip shapes can be distinguished. Arc (loose) chips occur when machining brittle materials, which fracture along the shearing surface. Such chip formation is accompanied by significant disruptions in the cutting force. Such chips take up little space and easily leave the machining zone. Segmented (discontinuous) chips are formed when machining hard materials with poor thermal conductivity. The high strength of the material causes shearing to occur rapidly in favored planes, without heat exchange with the surroundings. Stepped chip formation is accompanied by large fluctuations in cutting forces. Continuous (stringy) chips are formed when cutting ductile materials with a low yield strength. The variation in cutting forces when machining ductile materials is relatively small. These chips are the most difficult to remove and take up the most space.

**Figure 8:**
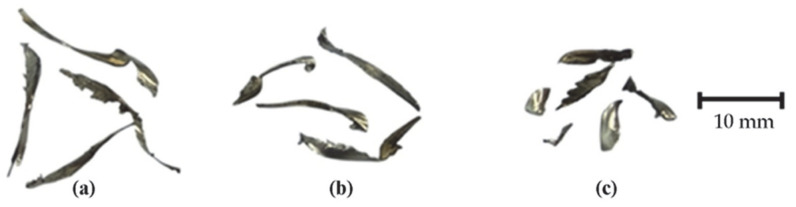
Shape of AISI 316L chips generated during drilling at the cutting speed of v_c_ = 15 m/min and at different axial feed rate values: (**a**) 0.1 mm/rev, (**b**) 0.15 mm/rev, and (**c**) 0.2 mm/rev.

Chip breakability is defined as the number of segments in 100 g of chips [55]. Breaking a chip into segments involves increasing the thickness of the cutting layer. However, the cross-sectional area of the cutting layer depends on the axial feed rate. Figure 8 shows the chip shape obtained during drilling of AISI 316L material. It was noted that the feed per tooth (f_z_) has a significant impact on chip shape. For feed rates in the range of 0.075–0.10 mm, chips are susceptible to breaking into small segments. As the feed rate increases within this range, the average chip length decreases from 20 mm to 3 mm. This may be due to the fact that increasing the feed rate increases the chip stiffness (the chip cross-sectional area increases). It can also be observed that the effect of cutting speed on chip shape and size is less significant compared to the effect of feed rate. Cutting speed does not significantly affect chip breakability, and chips of the same length are observed throughout the entire range of cutting speeds analyzed. It is worth mentioning that chips can become tangled around the two flutes of the drill when the feed per tooth is relatively low (0.05 mm). This chip tangling may result from the difficulty in smoothly removing long chips from the drill flutes.

**Figure 9:**
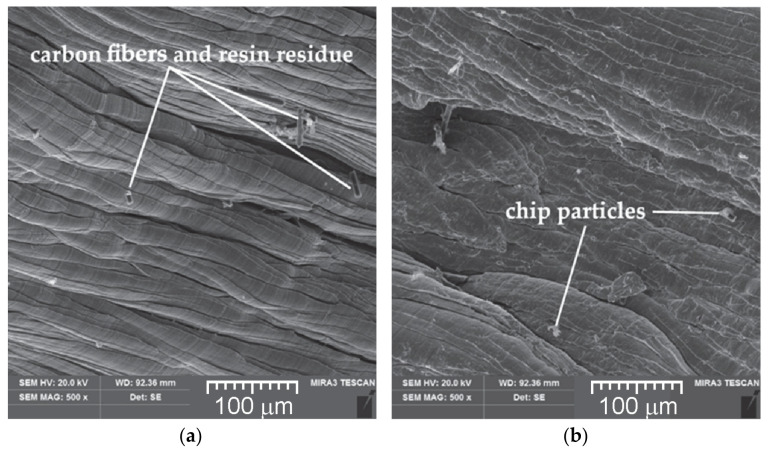
SEM micrographs of AISI 316L chips generated when drilling at a cutting speed of v_c_ = 15 m/min and a feed rate of f = 0.15 mm/rev for different drilling strategies: (**a**) CFRP → AISI 316L and (**b**) AISI 316L → CFRP.

It should be noted that chip breakability improves with increasing feed rate (in the analysed range) for both drilling strategies: CFRP → AISI 316L and AISI 316L → CFRP. However, when drilling with the AISI 316L → CFRP strategy, the AISI 316L chips are more regular and shorter. When drilling with the CFRP → AISI 316L strategy, the AISI 316L chips are subjected to compression and deformation to a much greater extent. Figure 9 shows the AISI 316L steel chip morphology for the two different drilling strategies. When drilling the CFRP layer first, carbon fibers and polymer matrix residues can be clearly observed adhering to the AISI 316L chips (Figure 9a). When drilling the AISI 316L steel plate first, no carbon fibers or polymer matrix residues are present on the surface of the steel chips (Figure 9b), indicating no scratches along the hole surface of the CFRP layer and a better chip removal process. When drilling CFRP/AISI 316L stacks from the CFRP layer side, it was observed that steel chips damaged the hole surface in the CFRP layer. Furthermore, the scratching action between the AISI 316L chips and the CFRP hole surface contributes to difficulties in chip flow through the drill’s spiral flute.

### 3.3. Characteristics of the Hole Surface Quality

**Figure 10:**
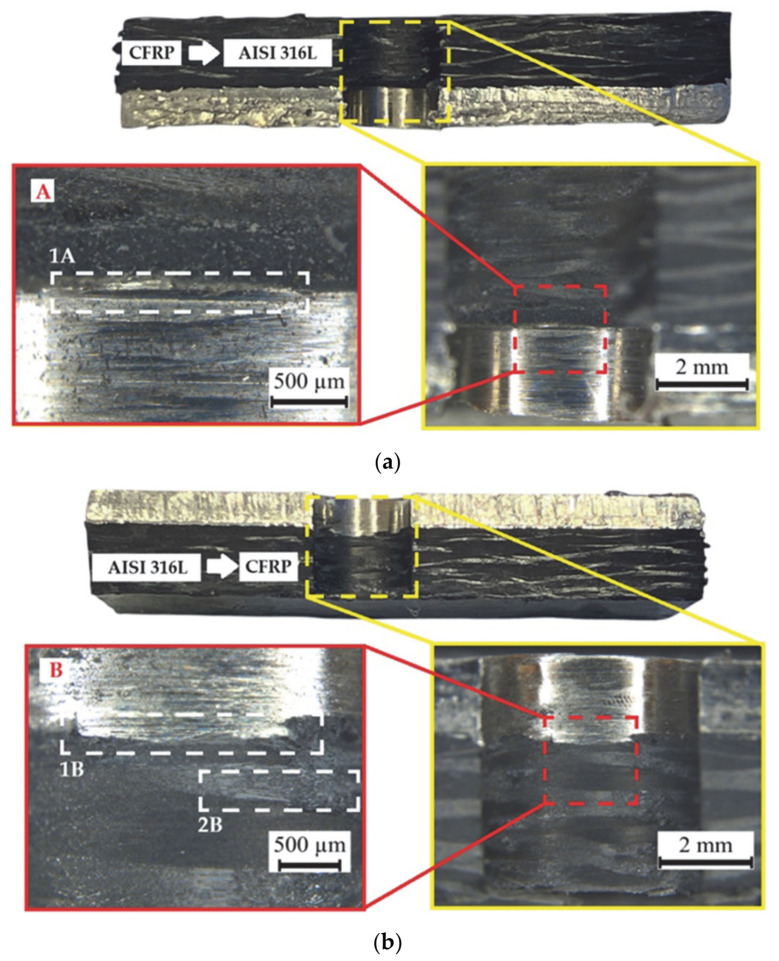
Hole surface damage when drilling at cutting speed v_c_ = 15 m/min and axial feed rate f = 0.15 mm/rev with different drilling strategies: (**a**) CFRP → AISI 316L and (**b**) AISI 316L → CFRP.

When drilling CFRP/AISI 316L stacks using different strategies, the hole surface is susceptible to damage, and the mechanisms causing damage are different. When drilling from the CFRP side, damage induced by AISI 316L chips is mainly caused by the difficult removal of chips from the machining zone. The stack interface between the CFRP and AISI 316L layers further blocks chip evacuation, leading to more severe mechanical damage to the hole surface, as shown in Figure 10a.

Furthermore, the hole surface at the interface is exposed to thermal damage caused by high-temperature steel chips. During drilling of the AISI 316L layer, the chips carry away most of the heat generated during the cutting process. Hot steel chips directly contact the hole surface in the CFRP layer, causing thermal damage to the CFRP hole surface. When drilling from the AISI 316L steel plate side, the chip removal process is smooth, but the drill bit temperature has a destructive effect on the CFRP layer. The drill bit’s temperature reaches approximately 192 °C (see Section 4). It can be observed in Figure 10b that the CFRP polymer matrix is locally burned where the drill bit started to penetrate into the CFRP layer area. This phenomenon indicates that the epoxy matrix was oxidized at high temperature [56]. The polymer matrix undergoes thermal degradation as it oxidizes, and this process significantly reduces the bond strength between the polymer matrix and the carbon fibers [57]. Cutting heat generated during drilling of the AISI 316L layer in the AISI 316L → CFRP drilling strategy is the main cause of damage to both the interface and the CFRP layer.

### 3.4. Hole Surface Topography

**Figure 11:**
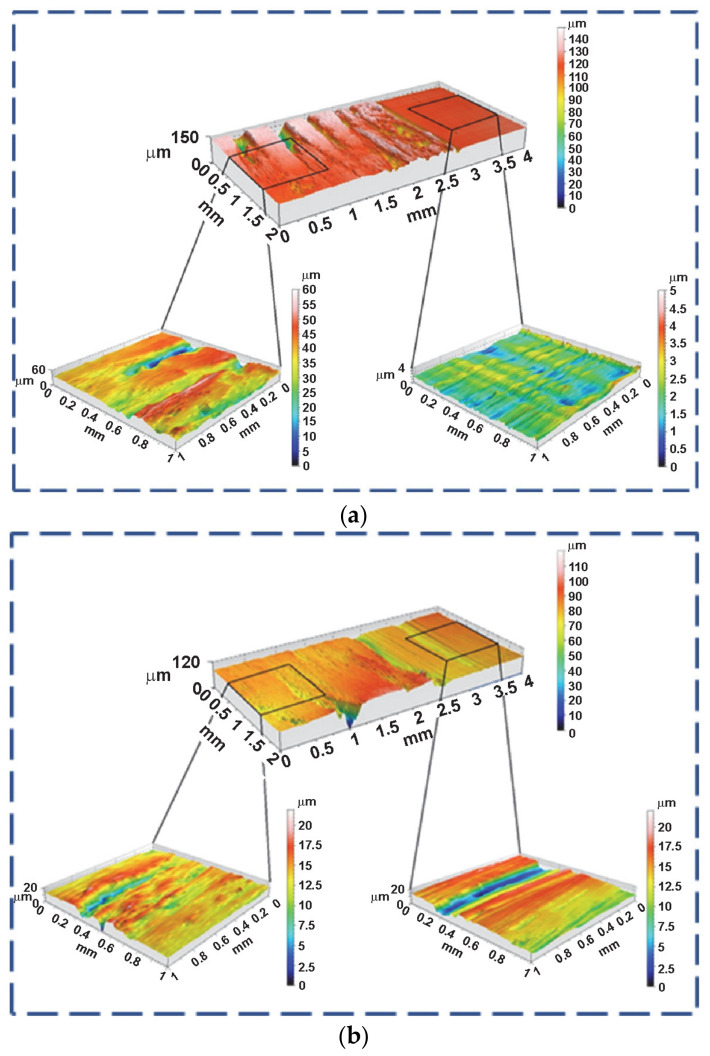
Hole surface topographies related to cutting speed v_c_ = 15 m/min and axial feed rate f = 0.15 mm/rev with different drilling strategies: (**a**) CFRP → AISI 316L and (**b**) AISI 316L → CFRP.

Hole surface topography is one of the key features considered when assessing surface quality in drilling processes. Figure 11 presents topographic maps of the hole cross-sectional surface for both drilling strategies analyzed. The surface topographies shown in Figure 11 were obtained by drilling at the cutting speed of 15 m/min and the axial feed rate of f = 0.15 mm/rev. Based on the surface topographic maps, selected surface roughness parameters (Table 3 and Table 4) were determined for the entire length of the drilled holes.

The surface topographies shown in Figure 11 clearly indicate different surface morphologies between the CFRP layer (3 mm thick) and the AISI 316L steel layer (1.5 mm thick). The conducted investigations of machined surface roughness confirmed the relationships commonly known from the literature and the authors’ previous studies regarding the effect of both cutting speed and axial feed rate on machined surface roughness. A tendency for the Sa parameter to decrease with increasing cutting speed and/or decreasing feed rate was observed.

**Figure 12:**
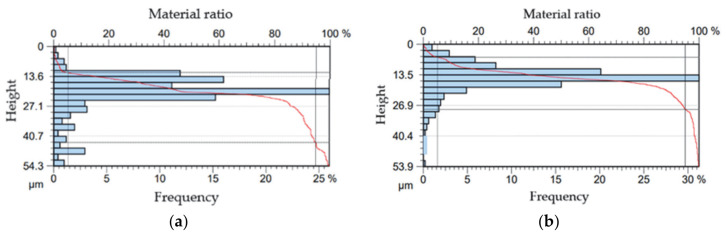
Bearing area curves of an obtained hole upon drilling at cutting speed v_c_ = 15 m/min and axial feed rate f = 0.15 mm/min with the strategy: (**a**) CFRP → AISI 316L and (**b**) AISI 316L → CFRP.

Another topographic feature analyzed was the Abbott–Firestone curve, which describes the surface texture of an object. Figure 12 shows bearing area curves (BACs) for borehole surfaces. The abscissa of the Abbott–Firestone curve represents the bearing capacity factor in percentage terms, while the ordinate axis represents the depth specified in micrometers. The Abbott–Firestone curve is used to describe the variation in profile or surface properties that change over the borehole surface. BAC shows the load length ratio curve determined as the cut level function. This is the cumulative probability density of the ordinate height of the surface profile.

Figure 12 shows the BAC for the borehole surface obtained at cutting speed v_c_ = 15 m/min and axial feed rate f = 0.15 mm/min. The hole surface for the AISI 316L → CFRP drilling strategy with the cutting parameters listed above is characterized by a less concentrated ordinate density distribution compared to the obtained hole upon drilling with the strategy CFRP → AISI 316L. For the surface shown in Figure 11a, 90% of the material is characterized by a core height Sk = 32.4 μm (Figure 12a). For the surface shown in Figure 11b, 90% of the material is characterized by a core height Sk = 24 μm (Figure 12b). The total height of the profile for holes drilled using both analyzed strategies is similar and is approximately 54 μm (Figure 12). Reduced peak height Spk accounts for asperities susceptible to rapid wear. Reduced peak height Spk for holes made with the drilling strategies CFRP → AISI 316L (Figure 12a) and AISI 316L → CFRP (Figure 12b) was 10.7 μm and 5.4 μm, respectively.

To analyze the quality of the machined surface, the arithmetic mean height Sa was used. It is a basic parameter of surface topography that defines the average absolute height of surface points relative to a reference plane. The arithmetic mean height Sa describes a surface more completely than the two-dimensional parameter Ra. The method for determining the Sa parameter takes into account the entire measurement area, resulting in a much more accurate representation of surface roughness, especially with irregular surface textures.

**Figure 13:**
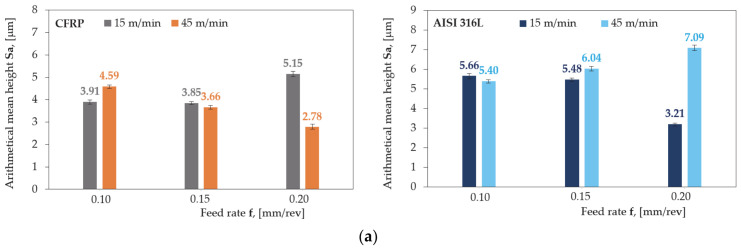

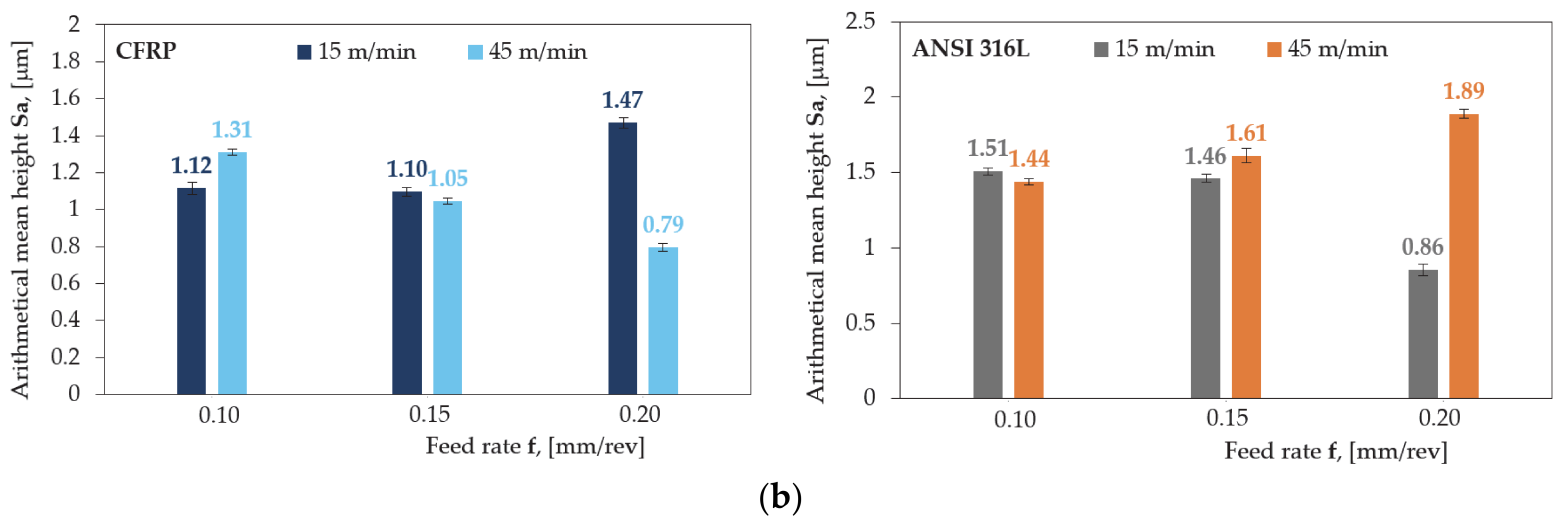
The effect of drilling process parameters on the arithmetical mean height Sa for the following drilling strategies: (**a**) CFRP → AISI 316L and (**b**) AISI 316L → CFRP.

Figure 13 presents the average arithmetic mean height Sa values for various drilling strategies. In the case of the CFRP → AISI 316L drilling strategy, it can be seen that the hole quality in the CFRP layer was the “worst” (Sa = 5.15 μm) at the lowest cutting speed of 15 m/min and the feed rate of 0.2 mm/rev (Figure 13a). In the case of the CFRP layer drilled at the cutting speed of 45 m/min, a decrease in the Sa parameter was observed as the axial feed rate increased. For the cutting speed of 45 m/min and the feed rate of 0.2 mm/rev, the arithmetical mean height Sa = 2.78 μm. However, for the cutting speed of 45 m/min and the feed rate of 0.1 mm/rev, the parameter Sa = 4.59 μm. An inverse relationship was observed when the cutting speed decreased. For the cutting speed of 15 m/min, a deterioration in surface roughness was observed as the feed rate increased. For the cutting speed of 15 m/min and the feed rate of 0.2 mm/rev, the arithmetical mean height Sa = 5.15 μm, and for the feed rate of 0.1 mm/rev, the arithmetical mean height Sa = 3.91 μm.

When drilling AISI 316L in the CFRP → AISI 316L drilling strategy, surface roughness deteriorated as the cutting speed increased and the feed rate simultaneously increased. At the cutting speed of 45 m/min and the axial feed rate of 0.2 mm/rev, the arithmetical mean height Sa = 7.09 μm, while at the cutting speed of 45 m/min and the feed rate of 0.1 mm/rev, the Sa parameter was 5.40 μm. A contrary relationship was observed for the cutting speed of 15 m/min; as the axial feed rate increased, the surface roughness improved. In the case of the cutting speed of 15 m/min and the axial feed rate of 0.2 mm/rev, the arithmetical mean height Sa = 3.21 μm; for the feed rate of 0.1 mm/rev, the Sa parameter is equal to 5.66 μm.

In the AISI 316L → CFRP drilling strategy, the hole quality was the “worst” (the highest value of Sa = 1.47 μm) for the CFRP laminate in the case of the lowest cutting speed of 15 m/min and the feed rate of 0.2 mm/rev. In the case of the CFRP laminate, as the cutting speed increased, a decrease in the Sa parameter was observed with a simultaneous increase in the axial feed rate value. For the cutting speed of 45 m/min and the feed rate of 0.2 mm/rev, the arithmetical mean height Sa = 0.79 μm; for the axial feed rate of 0.1 mm/rev, Sa is equal to 1.31 μm (Figure 13b). An inverse relationship was observed when the cutting speed decreased. For the cutting speed of 15 m/min and the feed rate of 0.2 mm/rev, the arithmetical mean height Sa = 1.47 μm, whereas for the axial feed rate of 0.1 mm/rev, the parameter Sa = 1.12 μm.

In the case of drilling in AISI 316L material for the CFRP → AISI 316L drilling strategy, as the cutting speed increased and the axial feed rate value simultaneously increased, an increase in the Sa parameter was observed (Figure 13b). For the cutting speed of 45 m/min and the axial feed rate of 0.2 mm/rev, the arithmetical mean height Sa = 1.89 μm, while for the axial feed rate of 0.1 mm/rev, the Sa parameter was 1.44 μm. For a cutting speed of 15 m/min and the feed rate of 0.2 mm/rev, the arithmetical mean height Sa = 0.86 μm, and for the feed rate of 0.1 mm/rev, the arithmetical mean height Sa was 1.51 μm.

### 3.5. Hole Surface Delamination in the CFRP Layer

**Figure 14:**
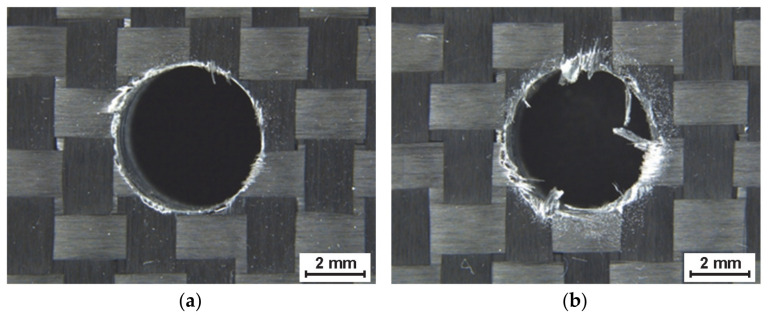
Delamination character of the hole surface drilled at v_c_ = 15 m/min and f = 0.15 mm/rev for the drilling sequence: (**a**) CFRP → AISI 316L and (**b**) AISI 316L → CFRP.

Drilling composite materials is a common process in many industries, as most components are assembled using riveted or bolted joints, and delamination is the leading cause of hole quality failure, accounting for 60% of all parts rejected due to failure of material processing [58]. The objective of this study was to determine the effect of cutting speed and axial feed rate on the occurrence of delamination during drilling of CFRP laminate. Figure 14 shows images of CFRP hole entry and hole exit after drilling of CFRP/AISI 316L stacks, depending on the drilling strategy.

The drilling strategy of the stacked layers has the greatest impact on CFRP laminate delamination. Relatively little delamination of holes in the CFRP layer was observed during drilling using the CFRP → AISI 316L strategy (Figure 14a) compared to the AISI 316L → CFRP drilling strategy (Figure 14b). This is due, among other things, to the effect of the AISI 316L plate supporting the CFRP laminate underneath during machining. When drilling from the AISI 316L side, the hole exit in the CFRP layer exhibits significant delamination defects. In both drilling strategies, the occurrence of delamination is dependent on the cutting parameters.

**Figure 15:**
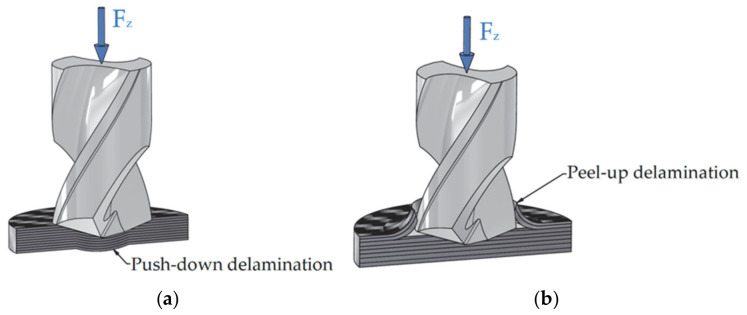
Types of delamination in drilling strategies (**a**) AISI 316L → CFRP and (**b**) CFRP → AISI 316L with cutting speed v_c_ = 15 m/min and axial feed rate f = 0.15 mm/rev.

The push-down delamination (Figure 15a) is caused mainly by the thrust force that the drill exerts on the material [16]. High axial feed rates, tool wear, and low interlaminar fracture toughness increase the risk of this type of damage. Push-down delamination is often considered more serious than peel-up delamination and can cause component rejection when drilling composite materials. Peel-up delamination (Figure 15b) is a type of mechanical damage that occurs where the drill enters the CFRP layer. It is characterized by the separation of the upper laminate layers as they are lifted by the helical flutes of the drill. Peel-up delamination is a consequence of the thrust force caused by the traction of the material by the drill [16,59]. If the thrust force (Fz) exceeds the adhesive bond strength between the layers, the CFRP delaminates. Higher axial feed rates are the primary cause of increased thrust force, which increases the likelihood of delamination. High cutting speeds generally reduce delamination because the cutting edges act more effectively to shear the fibers.

**Figure 16:**
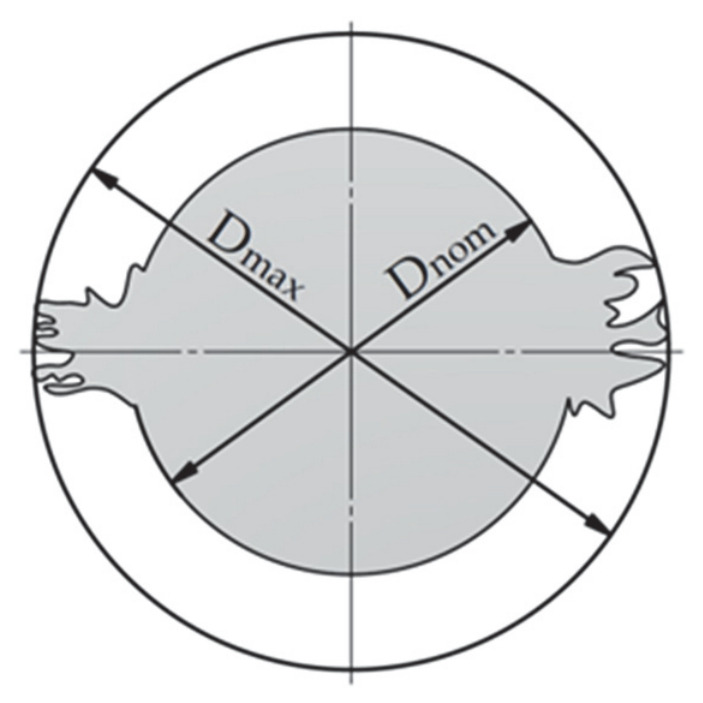
Schematic illustration of parameters used in the determination of the delamination factor F_d_.

During the literature review, contradictory effects of cutting speed on delamination were observed. Peel-up delamination was found to increase [60], decrease [61], or show a neutral trend [62] with increasing cutting speed. Similarly, push-down delamination was observed to increase [60,63], decrease [61,64,65], or remain essentially unchanged [66]. The phenomenon of push-down and peel-up surface delamination during drilling in CFRP, identified by Palanikumar [61], decreased in both drilling strategies after increasing the cutting speed. Obviously, the strongest influence on the character of delamination was observed in the aspect of the stack drilling strategy. The use of the CFRP → AISI 316L drilling sequence resulted in lower delamination factor F_d_ values. Figure 16 presents a graphical interpretation of the delamination factor F_d_.

The delamination factor F_d_ is the ratio of the delamination maximum diameter D_max_ to the nominal hole diameter D_nom_ [16]:

(1) $$F_{d}=\frac{D_{max}}{D_{nom}}$$

The delamination factor (F_d_) is the most commonly used criterion for assessing delamination damage in composite materials [67]. When drilling CFRP-based FMLs, the effect of drilling process parameters on delamination is a key aspect that has been studied over the years. The axial feed rate and cutting speed have an equally strong effect on delamination; therefore, lower feed rates and higher cutting speeds are recommended. Increasing cutting speed can lead to reduced delamination effects. Furthermore, drilling performance can be affected by the type of drill material and its coating [68]. In general, when drilling CFRP using a standard twist drill, a low axial feed rate combined with a high cutting speed can reduce the risk of delamination.

**Figure 17:**
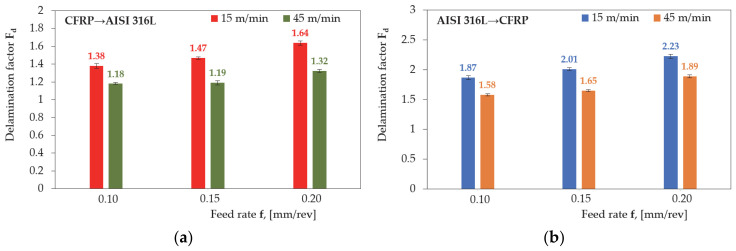
Influence of cutting parameters on the delamination factor F_d_-value for different drilling sequences: (**a**) CFRP → AISI 316L and (**b**) AISI 316L → CFRP.

Figure 17 shows the delamination factor (F_d_) as a function of cutting parameters for different drilling strategies. When drilling using the CFRP → AISI 316L strategy, the delamination factor increases with increasing axial feed rate and decreases with increasing cutting speed. At the cutting speed of 15 m/min and the feed of 0.1 mm/rev, the delamination factor is 1.38, and it increases to 1.64 (by 22%) at the feed rate of 0.2 mm/rev (Figure 17a). At the cutting speed of 45 m/min and the axial feed rate of 0.2 mm/rev, the delamination factor is 1.32, and it decreases by 12% (to F_d_ = 1.18) at the cutting speed of 45 m/min and the axial feed rate of 0.1 mm/rev. Similar to the CFRP → AISI 316L drilling strategy (Figure 17a), in the case of drilling with the AISI 316L → CFRP strategy, the delamination factor increases with increasing axial feed rate and decreases with increasing cutting speed (Figure 17b). At the cutting speed of 15 m/min and the axial feed rate of 0.1 mm/rev, the delamination factor is 1.87, and at the feed rate of 0.2 mm/rev, F_d_ = 2.23 (20% increase). At the cutting speed of 45 m/min and the axial feed rate of 0.2 mm/rev, the delamination factor is 1.89. Reducing the axial feed rate to 0.1 mm/rev reduces the delamination factor by 20% (to 1.58).

In industrial practice, two processes are required to ensure accurate hole drilling in CFRP/AISI 316L stacks: drilling and reaming. When using the CFRP → AISI 316L drilling strategy, although AISI 316L chips can easily cause severe scratches on the drilled hole surface in the CFRP layer, the reaming process can, in most cases, remove the resulting hole damage and improve the hole’s surface integrity. When using the AISI 316L → CFRP drilling strategy, although erosion damage to the CFRP layer caused by AISI 316L chips can be avoided, damage to the CFRP layer due to reaming cannot be completely removed. Furthermore, damage to the CFRP layer due to delamination is very sensitive to tool wear. Therefore, in most cases, the CFRP → AISI 316L drilling strategy is preferred in terms of minimizing delamination damage.

A qualitative analysis of the effect of cutting process parameters on the delamination factor was performed using analysis of variance (ANOVA). The ANOVA results for the delamination factor in CFRP → AISI 316L drilling are presented in Table 5. Both cutting speed and axial feed rate were significant parameters at a significance level of α = 0.05. The model F-value of 31.13 implies the model is significant. The overall coefficient of determination R^2^ is high (0.9540). The predicted R^2^ of 0.7880 is in reasonable agreement with the adjusted R^2^ of 0.9234 because the difference between these parameters is less than 0.2 (Table 6). The ANOVA model can be used to navigate the design space because adequate precision (the signal-to-noise ratio) is significantly greater than 4 (Table 6).

The final equation in terms of actual factors is as follows:

Delamination factor (CFRP → AISI 316L) = 1.330 − 0.008889 × v_c_ + 2.0 × f (2)

**Figure 18:**
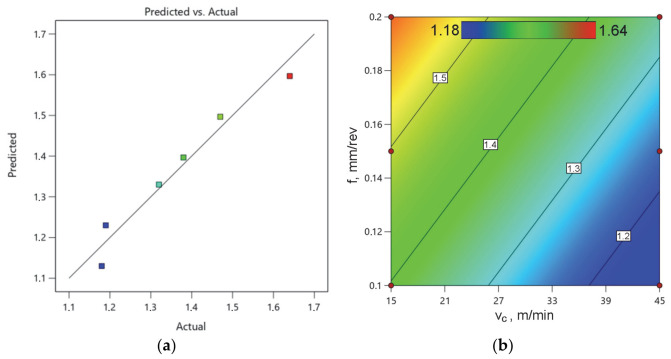
(**a**) Predicted versus actual response for the delamination factor and (**b**) response surface presenting the interaction between axial feed rate and cutting speed affecting the delamination factor in CFRP → AISI 316L drilling.

A comparison of the experimental values of the delamination factor in CFRP → AISI 316L drilling with the values predicted by the ANOVA model is presented in Figure 18a. The delamination factor values are evenly distributed around the line of perfect prediction. Delamination factor increases with decreasing cutting speed and with increasing axial feed rate (Figure 18b). High cutting speed values combined with small axial feed rate values determine low delamination factor values in the CFRP → AISI 316L drilling strategy.

The ANOVA results for the delamination factor in AISI 316L → CFRP drilling are presented in Table 7. Both cutting speed and axial feed rate were significant parameters at a significance level of α = 0.05. The model F-value of 63.51 implies the model is significant. The overall coefficient of determination R^2^ is high (0.9769). The predicted R^2^ of 0.9159 is in reasonable agreement with the adjusted R^2^ of 0.9615 because the difference between these parameters is less than 0.2 (Table 8). The ANOVA model can be used to navigate the design space because adequate precision (the signal-to-noise ratio) is significantly greater than 4 (Table 8).

The final equation in terms of actual factors is as follows:

Delamination factor (AISI 316L → CFRP) = 1.69917 − 0.011 × v_c_ + 3.35 × f (3)

**Figure 19:**
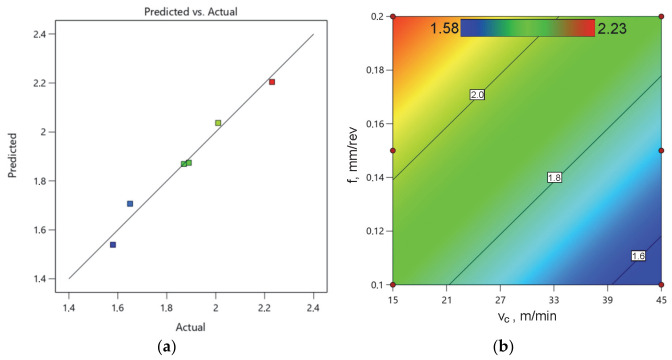
(**a**) Predicted versus actual response for the delamination factor and (**b**) response surface presenting the interaction between axial feed rate and cutting speed affecting the delamination factor in AISI 316L → CFRP drilling.

The delamination factor values for the AISI 316L → CFRP drilling strategy are evenly distributed around the line of perfect prediction (Figure 19a). The delamination factor increases with decreasing cutting speed and with increasing axial feed rate (Figure 19b), which is consistent with the results presented in Figure 18b. Small cutting speed values combined with large axial feed rate values determine high delamination factor values in the AISI 316L → CFRP drilling strategy.

**Figure 20:**
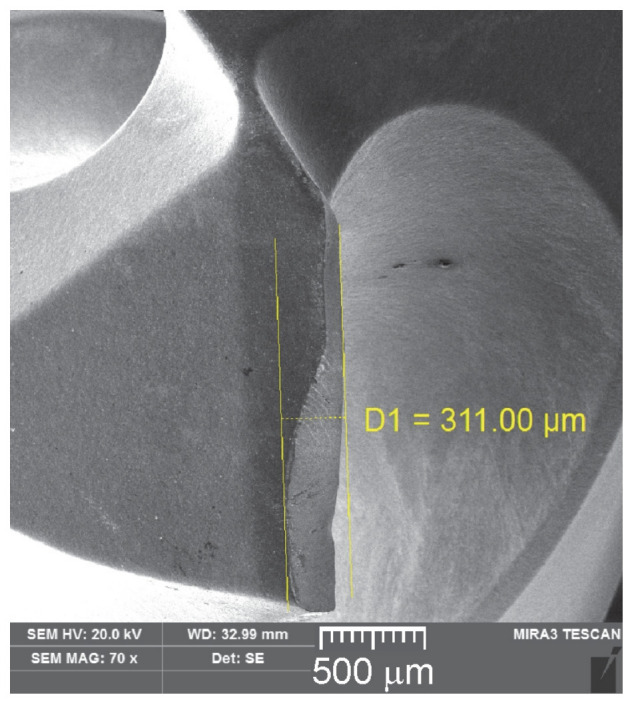
Maximum flank wear of the tool.

As mentioned in Section 3.2, each drill was used to drill only one hole to exclude the influence of tool wear on the hole damage during drilling. Therefore, the wear values of the drill edge were not measured during the experimental studies planned in this work. However, in the preliminary tests, drill wear was determined after drilling 30 holes at a rotational speed of 3000 rpm and an axial feed rate of 0.15 mm/rev. The wear value VB_max_ was 311 μm (Figure 20).

**Figure 21:**
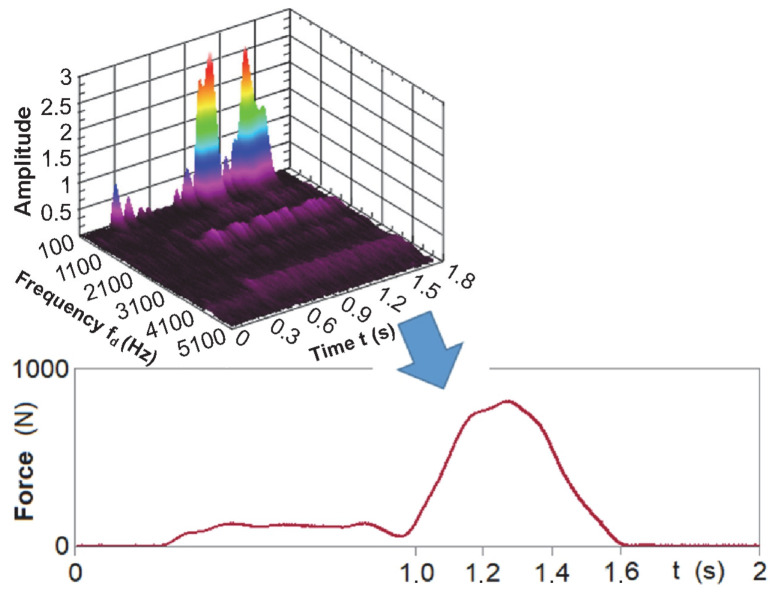
Spectrogram of the vibration signal and waveform of the signal of the thrust force; drilling process parameters: cutting speed v_c_ = 15 m/min and axial feed rate f = 0.15 mm/rev.

The recorded thrust force signals exhibit characteristic features associated with the transition from drilling in the CFRP layer to drilling in the AISI 316L layer when observed only in the time domain. However, upon analysis of the force signals in the frequency domain, a characteristic frequency range can be observed, in which an increased signal amplitude is the dominant feature, depending on the type of material being machined. Short-time Fourier transform (STFT) allows for the extraction of information from the signal about how its spectrum changes over time, i.e., simultaneous observation of its properties in both the time and frequency domains. The signal slice (sample block of size L) to be analyzed is divided into segments, and each segment undergoes an independent spectral analysis. Time window analysis allows for the localization of the signal’s spectral parameters in time. To obtain such frequency ranges, characteristic of the type of material being machined, short time intervals were applied using a window function, and the signal spectra were determined. The analysis assumed a Hanning window of 256 samples and an overlap of 128 samples. Figure 21 shows the force signal spectrum during drilling in various types of workpiece materials. The maximum amplitude of the thrust force signal in the frequency range of 0.1–5 kHz varies between 0.4 and 3.0 kHz, depending on the workpiece material. It can be seen that when drilling both CFRP and ANSI 316L layers, the signal spectrum is characterized by amplitude fluctuations at frequencies around 0.2–0.6 kHz. However, the amplitude value for the ANSI 316L material is more than five times greater than drilling the CFRP layer.

### 3.6. Burrs in AISI 316L Material

Burrs formed during drilling cause various assembly problems. Surface defects can reduce the fatigue life of parts because the work-hardened brittle burr can act as a crack initiation point [69]. One of the most important reasons to control burr formation during drilling is to minimize the total cost of the drilling operation, which can be expressed as the sum of the drilling (hole-making) costs and the deburring costs. Burrs are formed as a result of plastic deformation and crack propagation as the cutting tool blades exit the workpiece during the drilling process. It is estimated that up to 30% of typical component costs are due to deburring [55]. Burr morphology is determined by the magnitude of plastic deformation of the workpiece and the fracture mechanics [70]. Therefore, the burr formation mechanism is primarily determined by the properties of the workpiece material, in addition to the cutting parameters [71].

**Figure 22:**
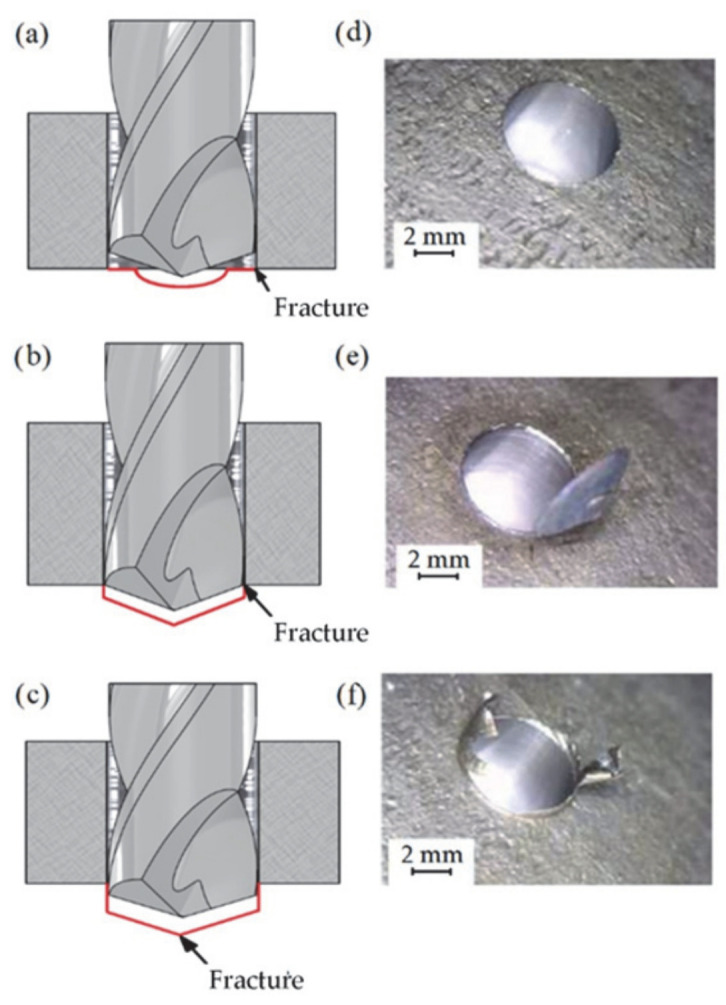
Location of a crack in the workpiece at the tool exit: (**a**) crack formed before the drill bits exit the hole, (**b**) crack formed along the exit edge of the hole, (**c**) crack formed after the drill bits exit the hole, (**d**) hole edge resulted from a crack formed before the drill bits exit the hole, (**e**) burr resulted from a crack formed along the exit edge of the hole, and (**f**) burr resulted from a crack formed after the drill bits exit the hole.

Three forms of burr formation were observed during drilling, as shown in Figure 22. As the drill bit approaches the exit of the workpiece, as shown in Figure 22a, plastic deformation of the remaining material increases. This remaining material can, therefore, be cut or pushed away by the thrust force. Under these drilling conditions, cracking is initiated at the edges of the drilled hole. Therefore, burr formation rarely initiates at the exit surface of the tool, as shown in Figure 22d. If plastic deformation continues after the drill bit exits the workpiece, two types of cracks can occur, as shown in Figure 22b,c. The first case concerns a crack formed along the exit edge of the hole, and the second case concerns a crack formed near the drill axis. In the first case, a cap (conical lid) is formed (Figure 22d), which can come off the part during the process or be easily removed afterwards [71]. If a crack starts near the drill axis, the cup may break into several pieces and remain as burrs, which are large and irregular, as shown in Figure 22f. The type of burr formed is determined by the initial fracture location of the material, which depends on the amount of plastic strain of the workpiece. The size of the burr also depends on the remaining cutting layer to be removed and the location of the crack initiation.

**Figure 23:**
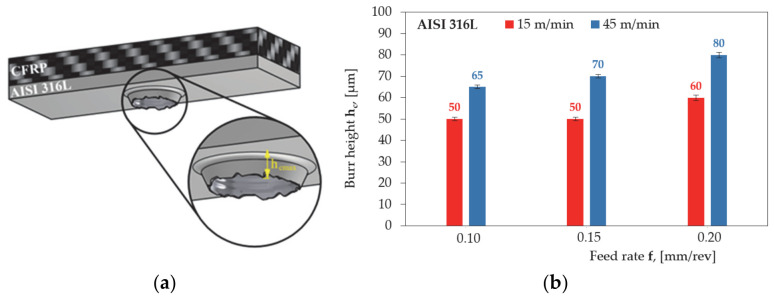
(**a**) Burr height measurement scheme and (**b**) the influence of cutting parameters on the burr height h_cmax_ for different drilling parameters.

Maximum burr height (h_cmax_) is commonly used to assess the surface quality of holes (Figure 23a). Figure 23b shows the variation of the maximum burr height with drilling parameters. The height of the burr uniformly distributed around the hole varies depending on the cutting parameters used. Because the initial fracture occurs in the central area of the hole and the material is then primarily pushed out rather than cut, the burr height depends on the ductility of the workpiece. The burr height varies within a narrow range of 50–80 μm. The study did not analyze burr height criteria that would clearly divide burrs into acceptable and unacceptable from a technological perspective. However, it was observed that a simultaneous increase in the cutting speed and the axial feed rate increases burr height.

As is well known, an increase in axial feed rate causes an increase in thrust force [55]. Both burr height and width show a good correlation with thrust force. Since this force is correlated with the volume of deformed material in front of the tool that forms the burr, it can be concluded that the burr height is significantly dependent on the cross-section of the cutting layer [72]. When drilling using the CFRP → AISI 316L strategy, the change in burr height (h_cmax_) increases with increasing axial feed rate and cutting speed. At the cutting speed of 15 m/min and the feed of 0.1 mm/rev, the burr height is 50 μm, while it increases to 60 μm at a feed of 0.2 mm/rev (20% increase). At the cutting speed of 45 m/min and the axial feed rate of 0.2 mm/rev, the burr height is 80 μm and decreases to 65 μm at the cutting speed of 45 m/min and the feed rate of 0.1 mm/rev (23% reduction).

A qualitative analysis of the effect of cutting process parameters on the burr height was performed using analysis of variance (ANOVA). The ANOVA results for the burr height drilling are presented in Table 9. Both cutting speed and axial feed rate were significant parameters at a significance level of α = 0.05. The model F-value of 36.58 implies the model is significant. The overall coefficient of determination R^2^ is high (0.9606). The predicted R^2^ of 0.8440 is in reasonable agreement with the adjusted R^2^ of 0.9343 because the difference between these parameters is less than 0.2 (Table 10). The ANOVA model can be used to navigate the design space because adequate precision (the signal-to-noise ratio) is significantly greater than 4 (Table 10).

The final equation in terms of actual factors is as follows:

Burr height = 25.41667 + 0.611111 × v_c_ + 125.0 × f (4)

**Figure 24:**
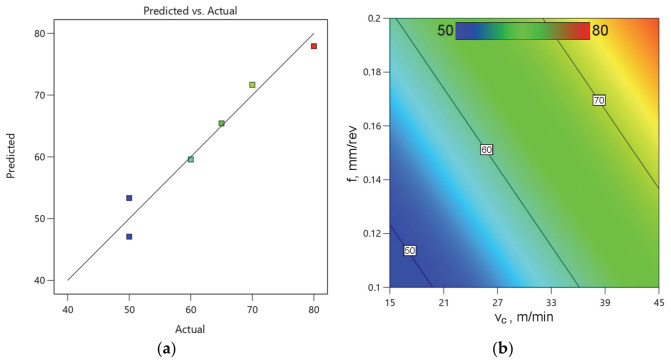
(**a**) Predicted versus actual response for the burr height and (**b**) response surface presenting the interaction between axial feed rate and cutting speed affecting the burr height in CFRP → AISI 316L drilling.

A comparison of the experimental values of the burr height in CFRP → AISI 316L drilling with the values predicted by the ANOVA model is presented in Figure 24a. The burr height values are evenly distributed around the line of perfect prediction. Burr height increases with both increasing cutting speed and axial feed rate (Figure 24b). High cutting speed values combined with high axial feed rate values determine high burr height values in the CFRP → AISI 316L drilling strategy.

This article examines the dry drilling process of CFRP/AISI316L stacks. Knápek et al. [73] performed drilling of CFRP systems without the use of cutting fluid (dry machining) to simulate a real industrial process. In common applications, dry machining is used in cutting CFRP plates [52,74], although such conditions increase tool wear and the risk of thermal damage to the polymer. However, Tashiro et al. [75] found longer tool life for dry machining than for water–mist–cooling conditions. Cutting fluids can penetrate the composite structure through damaged fibers and can impair the evacuation of metal chips mixed with CFRP dust [76]. The polymer matrix is also susceptible to moisture adsorption, which reduces fatigue life and complicates subsequent assembly and sealing processes. For this reason, dry drilling is dominant in the industry.

Tool material influences the workpiece–tool interaction governing hybrid stack drilling. Drills made of tungsten carbide are susceptible to increased abrasive wear and do not provide sufficient tool life when machining CFRPs [77]. Currently, increased tool life is achieved by applying protective coatings to the tool surface, such as TiAlN, TiAlN/TiN, AlCrN, AlCrSi/TiN, and others. As demonstrated in this work, the main problems with hole quality occur in the CFRP layer due to its non-homogeneous fiber structure. In this work, a TiAlN-coated drill, recommended for general use in a variety of operations with, among others, austenitic stainless steels, was used. In machining CFRP/AISI 316L stacks, the drill material must ensure effective cutting of both layers of the stack. Drilling of the AISI 316L layer is characterized by high temperatures and adhesion, while the CFRP layer is dominated by abrasive wear of the tool caused by carbon fibers. Under such conditions, TiAlN coatings are the optimal choice, characterized by good oxidation resistance and thermal stability, as well as high durability when machining difficult-to-machine materials [78]. Isbilir and Ghassemieh [79] found that TiAlN/TiN-coated WC-Co drills provide stable hole quality while limiting laminate delamination and tool wear. CFRP/Ti drilling studies [80] showed that TiAlN-coated drills provide longer tool life compared to AlTiSiN-coated tools. TiAlN coatings deposited via PVD were frequently reported for the drilling application of CFRP-Ti stacks [81]. TiAlN-coated drills offer a compromise between the adhesion resistance required when drilling AISI 316L steel and the wear resistance required when machining CFRP.

### 3.7. Temperature in Drilling

**Figure 25:**
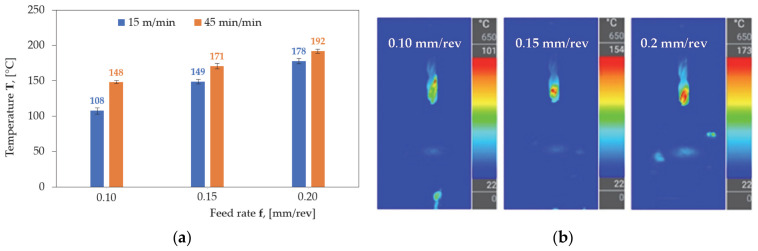
(**a**) Influence of drilling process parameters on chip temperature and (**b**) thermal images of chip temperature near the exit from the hole (cutting speed v_c_ = 15 m/min, AISI 316L → CFRP drilling strategy).

Temperature is an important factor to consider when drilling CFRP/AISI 316L stacks. Cured epoxy resin does not exhibit a typical melting point because it is a thermoset. Instead of melting, it degrades (burns) when heated. The glass transition temperature is the point at which the resin begins to soften and lose its mechanical properties. Above the glass transition temperature, the resin becomes plastic and ultimately decomposes (degrades), which typically occurs at around 400 °C [82]. As mentioned earlier, the chemical reaction temperature for the oxidation of epoxy resin in CFRP laminate is around 190 °C. The resin matrix will burn or degrade when the temperature induced by drilling exceeds the glass transition temperature, and the reduced bond strength between the resin and fibers will cause mechanical damage, such as fiber pullout and delamination. During the drilling using the CFRP → AISI 316L strategy, the cutting temperature in the CFRP layer gradually increases and stabilizes at 43 °C, which is lower than the glass transition temperature of the epoxy resin. No significant changes in the drilling temperature in the CFRP layer were observed. During the drilling using the AISI 316L → CFRP strategy, the cutting temperature increases rapidly and approaches or exceeds the glass transition temperature of the resin matrix. The maximum drilling temperature in the AISI 316L material was recorded as 192 °C (Figure 25a).

Figure 25b illustrates the chip temperature distribution for the cutting speed of 15 m/min and three axial feed rates. In all cases, it can be seen that the high temperature comes mainly from the AISI 316L chips due to their intense plastic deformation and cutting-induced friction. At the same cutting speed, the heat-affected zone is significantly larger for the higher axial feed rate (Figure 25b). The low thermal conductivity of the AISI 316L alloy causes a local increase in the temperature near the hole surface.

No significant effect of drilling sequence on temperature changes was observed. The temperature during drilling of the CFRP layer is significantly lower than when drilling AISI 316L material, and the maximum drilling temperature increases with increasing axial feed rate. Cutting speed was found to have a similar effect on temperature during drilling CFRP/AISI 316L stacks as axial feed rate.

## 4. Conclusions

This article presents the results of a pilot study of the drilling efficiency of CFRP/AISI 316L hybrid stacks. The effects of different drilling strategies and cutting parameters were investigated. Experiments have demonstrated the significant role of drilling strategy and cutting parameters in ensuring appropriate hole performance in CFRP/AISI 316L stacks. Based on the obtained results, the following conclusions can be drawn.

- In drilling CFRP/AISI 316L hybrid stacks, four cutting stages can be distinguished with respect to the interaction of the drill with the workpiece. The thrust force during drilling in the AISI 316L layer is higher for the CFRP → AISI 316L drilling strategy compared to the AISI 316L → CFRP drilling strategy. The reason for this is that the evacuation of ANSI 316L chips is hindered, which leads to an increase in the thrust force. Steel chips increase the friction between the drill and the hole surface drilled in the CFRP layer, leading to an increase in the thrust force.
- When drilling CFRP/AISI 316L hybrid stacks, both long and short AISI 316L chips were observed, depending on the cutting parameters. Higher axial feed rates resulted in short, segmented AISI 316L steel chips, which improved chip breakage. The effect of cutting speed on steel chip shape was insignificant.
- Both axial feed rate and cutting speed are important factors that influence hole surface quality, as defined by arithmetic mean height Sa. All tests showed that the Sa parameter for hole surfaces in AISI 316L and CFRP layers is largely dependent on the drilling strategy used. Lower Sa values were observed for CFRP drilling in the AISI 316L → CFRP strategy compared to the CFRP → AISI 316L strategy. Using a cutting speed of 45 m/min and feed rates in the range of 0.15–0.2 mm/rev increased Sa values for AISI 316L steel plate compared to a cutting speed of 15 m/min. The opposite trend was observed for drilling in the CFRP layer—increasing cutting speed decreased Sa values for the same axial feed rate. Regardless of the drilling strategy used, using lower cutting speed values reduces the surface roughness of the holes.
- Hole damage analysis indicates that drilling with the CFRP → AISI 316L strategy promotes lower delamination than drilling with the AISI 316L → CFRP strategy, under the same cutting parameters. This is because the AISI 316L plate acts as a stiffener in the stack as the drill exits the CFRP layer. It was confirmed that both axial feed rate and cutting speed play a key role in the development of drill-induced delamination. In terms of delamination minimization, drilling with the AISI 316L → CFRP strategy leads to an increased delamination factor at high axial feed rates and cutting speeds compared to drilling with the CFRP → AISI 316L strategy.
- Drilling using the AISI 316L → CFRP strategy favors the improvement of the hole surface quality (lower value of the Sa parameter) in the CFRP layer and, at the same time, favors the reduction in the burr size in the AISI 316L layer. Drilling using the CFRP → AISI 316L strategy only reduces the delamination factor value.
- Chip temperature during drilling the CFRP layer is significantly lower than during drilling the AISI 316L steel layer, and the maximum temperature during drilling CFRP/AISI 316L stacks increases with increasing axial feed rate and cutting speed. No significant effect of drilling strategy on temperature changes was observed. When using the CFRP → AISI 316L drilling strategy, damage to the hole surface (scratches) in the CFRP material caused by AISI 316L steel chips should be expected. In turn, the AISI 316L → CFRP drilling strategy can lead to heat-induced oxidation of the epoxy resin on the hole surface in the CFRP layer.

In summary, the AISI 316L → CFRP drilling strategy seems more reasonable for drilling CFRP/AISI 316L hybrid stacks. However, this drilling strategy increases the size of the delamination in the CFRP layer at the drill exit. This, in turn, necessitates the use of additional support under the CFRP layer when drilling using the AISI 316L → CFRP strategy to avoid damage caused by delamination at the hole exit.

The aim of the research presented in this article was to determine the values and temperature changes of AISI316L chips and tools (depending on cutting parameters) that may be in direct contact with the CFRP hole surface. It can also be concluded that there is a significant difference between the thermal damage area and the heat-affected area, which is determined by exceeding the glass transition temperature of the resin matrix. The heat-affected area is larger than the thermal damage area. Future research is planned to measure the thermal damage area of CFRP based on the area of hole surface discoloration [83] and using thermocouple wires mounted in the drill channel [83]. Hole–wall temperature and interface temperature will better support thermal damage analysis. To thoroughly verify the relationship between the discoloration area and the thermal damage area, measurements and analyses of the elastic modulus of the CFRP resin matrix are planned for selected measurement points. Dynamic mechanical analysis (Dynamic Mechanical Analysis/Dynamic Mechanical Thermal Analysis) will be used to precisely determine the material’s behavior under temperature changes, as well as nanoindentation tests to determine the material’s hardness and indentation modulus on a cross-section of the composite sample. Based on the obtained results, it will be possible to determine whether the elastic modulus of the resin matrix in the discolored area of the surface layer is lower than in the undiscolored area. Therefore, it will be possible to determine whether the area of thermal damage of CFRP can be approximately determined based on discoloration. However, it can be concluded that there is a significant difference between the thermal damage area and the heat-affected area, which is defined by exceeding the glass transition temperature of the resin matrix.

## Figures and Tables

**Table 1:** Selected mechanical properties of CFRP and AISI 316L materials.

Material	Tensile Strength (MPa)	Elongation (%)	Density (g/cm^3^)	Elastic Modulus (GPa)	Rockwell Hardness	Thermal Conductivity (W/(m·K))
ANSI 316L	485	40	7.9 (0.7)	200	95	14.6
CFRP	2620	7	1.55 (0.3)	65	91	~1

**Table 2:** Machining conditions.

Drilling Parameters	Level
Cutting speed v_c_ (m/min)	15, 45
Axial feed rate f (mm/rev)	0.10, 0.15, 0.20
Drilling strategy	CFRP → AISI 316L, AISI 316L → CFRP

**Table 3:** Selected parameters of hole surface roughness.

Drilling Strategy	Sq, μm	Ssk	Sku	Sp, μm	Sv, μm	Sz, μm	Sa, μm
CFRP → AISI 316L	13.50	−2.99	18.60	30.70	119	150	7.65
AISI 316L → CFRP	7.73	−1.93	13.30	39.80	80.50	120	4.97

**Table 4:** Basic functional parameters from the Abbott–Firestone curves.

Drilling Strategy	Parameters of the Abbott-Firestone Curve				
	Rpk, μm	Rvk, μm	Rk, μm	Mr1, %	Mr2, %
CFRP → AISI 316L	11.90	11.70	31.80	5	95
AISI 316L → CFRP	5.67	10.60	23.10	5	95

**Table 5:** ANOVA results of the delamination factor in CFRP → AISI 316L drilling (DF—degree of freedom).

Source	Sum of Squares	DF	Mean Square	F-Value	*p*-Value	Assessment
Model	0.1467	2	0.0733	31.13	0.0099	significant
v_c_	0.1067	1	0.1067	45.28	0.0067	
f	0.0400	1	0.0400	16.98	0.0259	
Residual	0.0071	3	0.0024			
Correlation total	0.1537	5				

**Table 6:** Fit statistics for ANOVA of the delamination factor in CFRP → AISI 316L drilling.

Standard Deviation	Mean	Coefficient of Variance, %	Coefficient of Determination R^2^	Adjusted R^2^	Predicted R^2^	Adequacy Precision
0.0485	1.36	3.56	0.9540	0.9234	0.7880	13.5980

**Table 7:** ANOVA results of the delamination factor in AISI 316L → CFRP drilling (DF—degree of freedom).

Source	Sum of Squares	DF	Mean Square	F-Value	*p*-Value	Assessment
Model	0.2756	2	0.1378	63.51	0.0035	significant
v_c_	0.1634	1	0.1634	75.30	0.0032	
f	0.1122	1	0.1122	51.73	0.0055	
Residual	0.0065	3	0.0022			
Correlation total	0.2821	5				

**Table 8:** Fit statistics for ANOVA of the delamination factor in AISI 316L → CFRP drilling.

Standard Deviation	Mean	Coefficient of Variance, %	Coefficient of Determination R^2^	Adjusted R^2^	Predicted R^2^	Adequacy Precision
00466	1.78	2.49	0.9769	0.9615	0.9159	20.1912

**Table 9:** ANOVA results of the burr height in CFRP → AISI 316L drilling (DF—degree of freedom).

Source	Sum of Squares	DF	Mean Square	F-Value	*p*-Value	Assessment
Model	660.42	2	330.21	36.58	0.0078	significant
v_c_	504.17	1	504.17	55.85	0.0050	
f	156.25	1	156.25	17.31	0.0253	
Residual	27.08	3	9.03			
Correlation total	687.50	5				

**Table 10:** Fit statistics for ANOVA of the burr height.

Standard Deviation	Mean	Coefficient of Variance, %	Coefficient of Determination R^2^	Adjusted R^2^	Predicted R^2^	Adequacy Precision
3.00	62.50	4.81	0.9606	0.9343	0.8440	14.5126

## Data Availability

The original contributions presented in this study are included in the article. Further inquiries can be directed to the corresponding author.
